# Thermal Study of Polyols for the Technological Application as Plasticizers in Food Industry

**DOI:** 10.3390/polym10050467

**Published:** 2018-04-25

**Authors:** Alberto Toxqui-Terán, César Leyva-Porras, Miguel Ángel Ruíz-Cabrera, Pedro Cruz-Alcantar, María Zenaida Saavedra-Leos

**Affiliations:** 1 Doctorate Institutional in Engineering and Materials Science (DICIM), Sierra Leona 530, Lomas, 2nd Section, C.P. 78210 San Luis Potosí, Mexico; alberto.toxqui@cimav.edu.mx; 2 Advanced Materials Research Center (CIMAV), Alianza Norte 202, Research and Technological Innovation Park (PIIT), C.P. 66600 Apodaca, Mexico; 3 Advanced Materials Research Center (CIMAV), Miguel de Cervantes # 120, Complejo Industrial Chihuahua, C.P. 31136 Chihuahua, Mexico; cesar.leyva@cimav.edu.mx; 4 Materials Research Center (DIP-CUCEI), Universidad de Guadalajara, Av. Revolución # 1500, Col. Olímpica, C.P. 44430 Guadalajara, Mexico; 5 Faculty of Chemistry Sciences, Autonomous University of San Luis Potosí, Manuel Nava 6, C.P. 78290 San Luis Potosí, Mexico; mruiz@uaslp.mx; 6 Academic Coordination, Altiplano Region, Autonomous University of San Luis Potosí, Road Cedral km. 5+600, C.P. 78700 Matehuala, Mexico; pedro.cruz@uaslp.mx

**Keywords:** polyols, thermal properties, glass transition temperature, devitrification temperature, modulated differential scanning calorimetry (MDSC)

## Abstract

In this work is presented the complete thermal analysis of polyols by direct methods such as simultaneous thermogravimetric and differential thermal analyzer (TGA-DTA), differential scanning calorimetry (DSC), modulated DSC (MDSC), and supercooling MDSC. The different thermal events in the temperature range of 113–553 K were identified for glycerol (GL), ethylene glycol (EG), and propylene glycol (PG). Boiling temperature (T_B_) decreased as GL > EG > PG, but increased with the heating rate. GL showed a complex thermal event at 191–199 K, identified as the glass transition temperature (T_g_) and devitrification temperature (T_dv_), and a liquid–liquid transition (T_L-L_) at 215–221 K was identified as the supercooling temperature. EG showed several thermal events such as T_g_ and T_dv_ at 154 K, crystallization temperature (T_c_) at 175 K, and melting temperature (T_m_) at 255 K. PG also showed a complex thermal event (T_g_ and T_dv_) at 167 K, a second devitrification at 193 K, and T_L-L_ at 245 K. For PG, crystallization was not observed, indicating that, during the cooling, the liquid remained as an amorphous solid.

## 1. Introduction

In the field of food products, comestible films have been well accepted in applications related to the extension of the shelf life of products [1]. This type of films is employed for conserving the quality and stability of products, acting as a selective transfer barrier for environmental gases and moisture, preventing the degradation of nutrients and the loss of volatile compounds responsible of imparting specific properties to the product [2]. Nowadays, different materials are employed in the production of comestible films. These materials are typically based in biopolymers such as polysaccharides, cellulose, starch, gums, carrageenan and alginate [3]. However, it is often necessary to add a plasticizer to extend the flexibility, and avoid the fracture of the film during handling. Plasticizers are low molecular weight, and volatile compounds widely employed in the polymer industry as additives [3]. By definition, a plasticizer is a substance incorporated into a rigid material used for increasing the flexibility, and compliance [4]. Commonly, polar molecules used as plasticizers are intercalated within the polymeric chains, modifying the hydrogen bond interactions and increasing the free volume of the matrix material [4]. Consequently, the morphological, mechanical, and thermal properties such as strain, hardness, density, viscosity, and glass transition temperature may be modified [1,5,6,7]. The plasticizers most commonly employed as additives in the preparation of comestible films include the family of polyols, and some sugars with similar properties to polyols [8,9,10]. In biopolymers, the plasticizer often found is water [11]. However, the main disadvantage of water is its rapid evaporation even at standard temperature and pressure conditions. In some food products, small variations in the water content may lead to notorious changes in the final state of the product [12]. Thus, some other low molecular weight, and less volatile, molecules have been proposed as potential plasticizers for biopolymers. The family of polyols comprises hydroxylated organic compounds found in a wide range of molecular weights, such as glycerol (GL), sorbitol, ethylene glycol (EG), propylene glycol (PG), poly(ethylene) glycol (PEG), and poly(propylene) glycol (PPG) [13]. Polyols may improve the mechanical properties, water resistance, and aging of thermoplastic biopolymers, and food products [14,15,16,17,18]. Particularly, polyols are employed in applications where products must remain stored at low temperatures because they help to prevent the freezing of the product. At temperatures below the freezing point of water, some non-common thermal events such as vitrification (T_v_) and supercooling (T_co_) are observed in this family of plasticizers. The application field of polyols is extensive. For example, GL is the plasticizer most often used in the production of thermoplastic biopolymers such as starch [19,20,21]. The mixtures containing GL present a homogeneous, and soft morphological aspect, where the content of GL influences the mechanical properties of the film [21,22]. Besides, the use of sorbitol as sweetener in dietary products, the tensile strength of the product increases with the relative content of the plasticizer [23]. EG is an odorless synthetic liquid, hygroscopic and sweet to taste, mainly employed as antifreeze, and hydraulic fluid, but also used as plasticizer at low concentrations in food products [24]. PG is effectively used as crystallization modifier, humectant, softening agent, solvent, viscosity controller, and as aid in the rehydration of food products [25].

The behavior of polyols may be understood through the study of their thermal properties. Properties such as glass transition temperature (T_g_), crystallization (T_c_), melting (T_m_) and boiling (T_B_), allow determining the final state of the plasticizer, and thus predicting the state of the food product. The thermal properties of GL, EG and PG have been previously reported at a wide range of temperatures [26,27,28,29,30]. The direct thermal characterization techniques most commonly employed in these reports are differential scanning calorimetry (DSC), thermogravimetric analysis (TGA), modulated DSC (MDSC), rheological tests, and dynamic mechanical analysis (DMA) [29,31,32,33]. Indirect techniques include nuclear magnetic resonance (NMR) and fluorescence spectroscopies [28]. Takeda et al. [27] determined the T_g_ of polyols by quenching the samples in liquid nitrogen at 113 K, and the subsequent thermal analysis by DSC. They found T_g_ values of 186, 152 and 166 K for GL, EG and PG, respectively. Recently, Sou et al. [29] studied the thermal properties of GL employing laboratory-made equipment. They reported a T_g_ of 180 K, T_c_ of 205 K, a solid–solid transition (T_S-S_) of 229 K, and T_m_ of 289 K. Besides the differences in the values reported for the same thermal event, there are other physicochemical events detected and identified as an anomalous behavior near the T_g_ value [34].

Despite the extensive use of polyols as plasticizers, a complete study of their thermal properties where the different thermal events have been differentiated accurately has not yet been performed. Therefore, in this work are reported the thermal properties of three polyols: GL, EG and PG. The different thermal events were determined by direct thermal analysis (TGA, DSC, MDSC, and supercooling MDSC) in the temperature range of 113–553 K. The experiments were repeated at different heating rates with the aim of observing the displacement of the thermal events. With these results, it was possible to accurately identify the different thermal events, and relate them with the physicochemical changes of polyols.

## 2. Materials and Methods

### 2.1. Materials

Reagent grade glycerol (99.8%, Fermont, Mexico), ethylene glycol (99.9%, Fermont, Mexico) and propylene glycol (99.5%, Hycel, Mexico) were employed as received without further treatment.

### 2.2. Thermogravimetric (TGA), and Differential Thermal Analysis (DTA)

Thermogravimetric, and differential thermal analysis were carried out in a simultaneous TGA-DTA SDT Q600 (TA Instruments, New Castle, DE, USA). For DTA, baseline was calibrated with Indium (melting temperature of 429 K, and melting enthalpy of 28.47 (J/g)). Polyol samples ranging 29–32 mg were placed in 110 µL platinum crucibles. Thermograms were recorded at different heating rates (275, 278 and 283 K/min) over a temperature range of 298–573 K. Using Universal Analysis 2000© software (New Castle, DE, USA) the different features from the curves were identified: mass loss (wt %), initial, peak, and final melting temperatures (T_m onset_, T_m peak_ and T_m endset_, respectively); and initial, peak and final boiling temperatures (T_B onset_, T_B peak_ and T_B endset_, respectively).

### 2.3. Differential Scanning Calorimetry (DSC) and Modulated DSC (MDSC)

A modulated DSC Q200 (TA Instruments, New Castle, DE, USA) equipped with an RCS90 cooling system was employed for determining the phase transitions of polyol samples. Instrument was calibrated with indium for melting temperature, and enthalpy; meanwhile, sapphire was used as the standard for the heat capacity (C_p_) calibration. Samples of 29–32 mg were encapsulated in Tzero^®^ aluminum pans (TA Instruments, New Castle, DE, USA), and kept under an isothermal condition of 178 K during 5 min. Thermograms were acquired at heating rates of 275, 278 and 283 K/min over a temperature range of 183–573 K. Thermograms were analyzed using the Universal Analysis 2000© software (TA Instruments, New Castle, DE, USA) and glass transition (T_g_), vitrification (T_v_), crystallization (T_c_) and melting (T_m_) temperatures were determined.

Modulated DSC experiments were carried out in the same equipment, and under the same experimental conditions as regular DSC measurements. The only difference was in the properties of the heating flux, which was set with temperature amplitude of ±274 K and a period of 40 s. All tests were done by triplicate.

### 2.4. MDSC at Supercooling Temperature (SMDSC)

A modulated DSC Q2000 (TA Instruments, New Castle, DE, USA) equipped with a liquid-nitrogen cooling system (LNCS) was employed for determining the phase/state transitions of polyols at the supercooling temperature. Instrument was also calibrated with indium and sapphire. Samples of about 8–19 mg were encapsulated in Tzero^®^ aluminum pans (TA Instruments, New Castle, DE, USA). Thermograms were acquired in the temperature range of 113–323 K at a heating rate 278 K/min. A temperature amplitude of ±274 K with a period of 40 s, and isothermal at 113 K by 5 min was used as the thermal procedure. The T_g_, T_v_, T_m_, and T_c_ values; solid–solid (T_s-s_) and liquid–liquid (T_L-L_) transitions; and devitrification temperatures (T_dv_) were identified from the thermograms. Each experiment was repeated three times.

## 3. Results and Discussion

### 3.1. Simultaneous Thermal Analysis (TGA-DTA)

**Figure 1:**
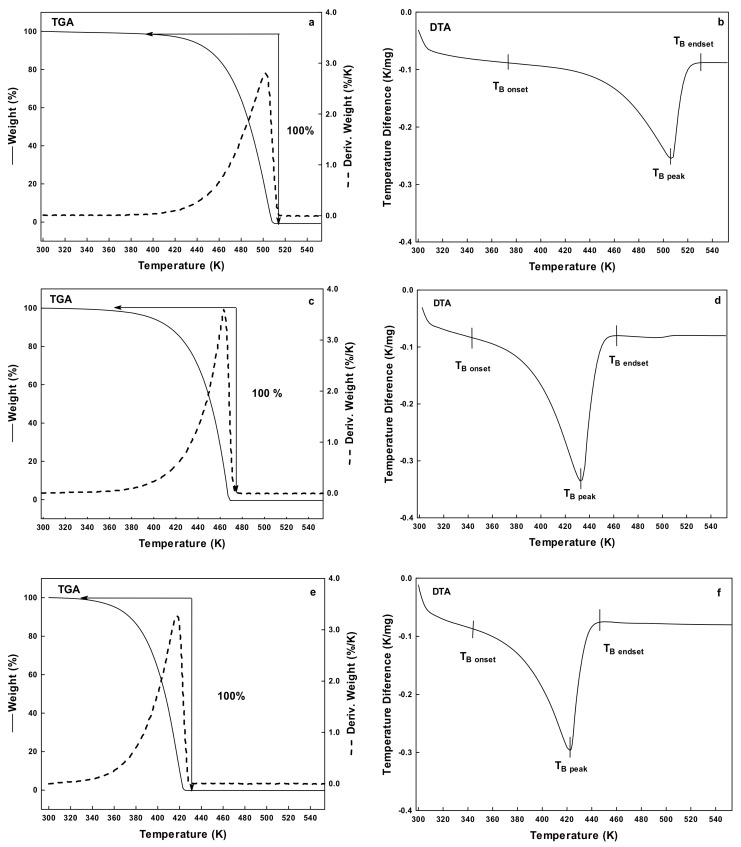
Simultaneous TGA-DTA results at a heating rate of 278 K/min: (**a**,**b**) GL; and (**c**,**d**) EG; and (**e**,**f**) PG. On the left column are plotted the mass loss curve (wt %) (continuous line), and the derivative of mass loss with temperature (%/K) (dotted line). On the right column is plotted the corresponding DTA curve.

TGA allows distinguishing physical, and chemical phenomena presented during the heating of a material. Specifically, it is possible to evaluate both thermal stability and degradation processes. The DTA measures the difference in temperature between the sample, and a reference material. With the simultaneous TGA-DTA, it is possible to determine whether a thermal event such as the weight loss is caused by an exothermic or endothermic process, and relate this observation with a chemical or physical change in the sample. Through this technique, the T_B_ of polyols was determined. Figure 1 shows the simultaneous TGA-DTA results at a heating rate of 278 K/min for the polyols studied in this work. The TGA curve (Figure 1a,c,e) showed the initial weight loss in a temperature of 373, 331, and 325 K, for GL, EG, and PG, respectively. In all cases, sample was brought to the total mass loss, and TGA curve showed a single-step weight loss behavior. The derivative of mass loss with respect to temperature (%/K) confirmed the presence of one-single thermal event, and indicated the final temperature for this event at 513, 437, and 431 K, for GL, EG and PG.

Figure 1b,d,f shows the corresponding DTA curve. The three polyols presented a similar endothermic curve, corresponding to the physical change of liquid into gas, named boiling temperature (T_B_). In liquids, boiling or evaporation is presented in a range of temperatures rather than in single temperature. Dou et al. [35] studied the thermogravimetric decomposition of crude and pure GL at multiple heating rates. They observed the decomposition (pyrolysis) of pure GL in the range of 422–552 K with a mass loss of about 95%. The results reported in this work agree with those values reported by Duo et al. However, based on the simultaneous TGA-DTA results, the identification of the thermal event corresponded to the evaporation of liquids rather than to the decomposition as indicated by Duo et al. Conversely, Agarwal and Lattimer [36] reported the complete evaporation of GL and EG as the total mass loss with a single endothermic peak. They also discussed the effect of heating rate on the evaporation behavior of polyols, showing a shift towards higher temperatures as heating rate was increased. Thus, in this work, the simultaneous TGA-DTA experiments were repeated at different heating rates (275, 278, and 283 K/min). Results are summarized in Table 1. Overall, polyols increased their boiling temperature range as heating rate was increased. These results were useful for setting the boiling temperature range, and the maximum temperature at which the polyols may be employed in a given application.

### 3.2. DSC, MDSC, and S-MDSC Analysis

DSC analysis is used for determining the phase transitions or conformational changes involving an energy change. These changes are induced by the temperature variation on the sample, and include the glass transition temperature, and melting. On the other hand, the MDSC analysis is employed for differentiating the various thermal events that may occur at the same temperature interval. The main difference between DSC and MDSC relies in the way sample is heated. While in DSC heating is supplied linearly at a desired rate (K/min), in MDSC the heating is modulated by a sinusoidal heat wave. The properties of the wave i.e., amplitude (temperature) and frequency (time) can be adjusted to properly distinguish, and separate the thermal events. Thus, the different thermal events may be separated into reversible, which depend on the heat capacity, and non-reversible, which depend on temperature and time. MDSC has been widely employed in the thermal characterization of biopolymers with complex thermal events [37,38,39]. Additionally, the S-MDSC employs a wider range of temperature for testing the sample, i.e., from 93 K to 833 K. With these techniques, it is possible to identify the different thermal events such as T_g_, T_c_, T_B_, T_v_, and others non-commonly reported such as the devitrification temperature (T_dv_), and solid–solid (T_S-S_), and liquid–liquid transitions (T_L-L_). The three techniques were employed in the determination of the different thermal events of the polyols studied in this work. Except where noted, all results were obtained at a heating rate of 278 K/min.

**Figure 2:**
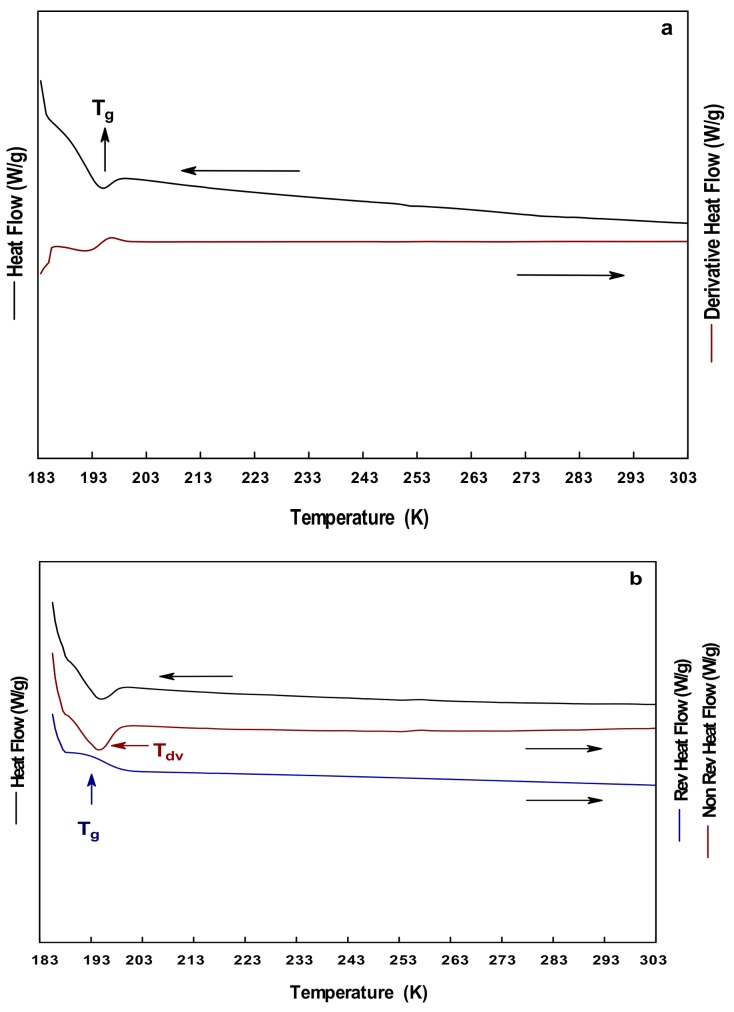

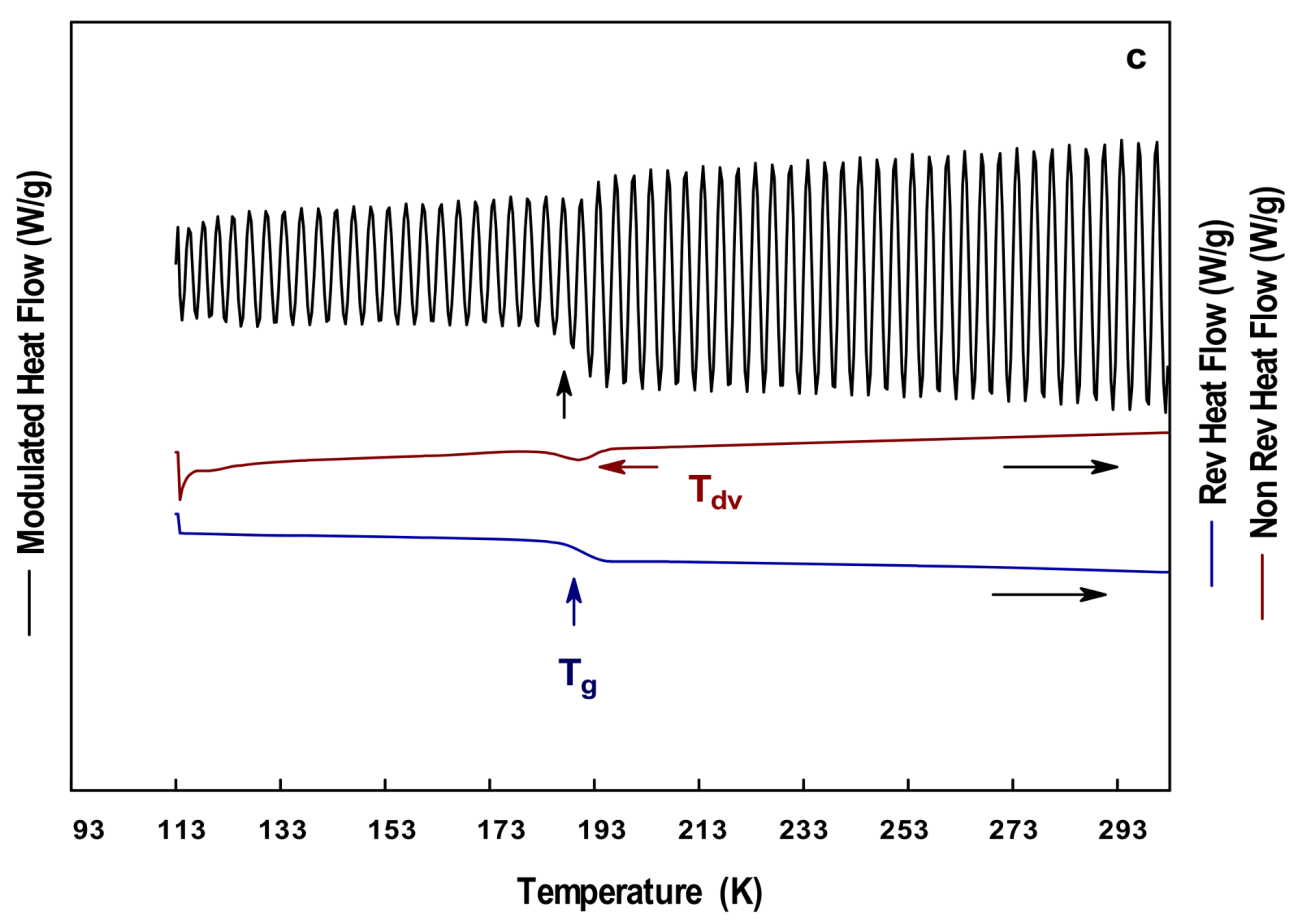
Thermograms of GL at 278 K/min: (**a**) DSC; (**b**) MDSC; and (**c**) SMDSC.

**Figure 3:**
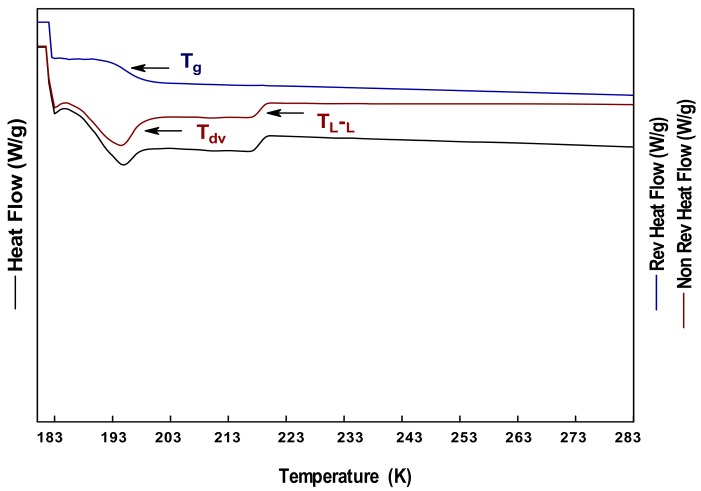
MDSC thermograms of GL at 275 K/min.

Figure 2a–c shows the thermograms obtained from DSC, MDSC, and S-MDSC for the GL. The total heat flow curve showed an endothermic peak in the range of 193–203 K (Figure 2a,b). The separation of the heat flow curves from the MDSC (Figure 2b) allowed properly differentiating the thermal events. The reversible heat flow curve showed a stepped change in the slope identified as the T_g_ of GL, in the range of 191–199 K. The non-reversible heat flow curve showed an endothermic peak in the same temperature range, which was identified as the T_dv_ with a value of 197 K. These observations were confirmed from the S-MDSC results (Figure 2c), where no other thermal events were observed at the heating rate of 278 K/min, and in the temperature range of 113–298 K. Figure 3 shows the MDSC thermograms of GL at 275 K/min, where a stepped change in the slope of the non-reversible flow curve was observed, and identified as the liquid–liquid transition (T_L-L_) or supercooling temperature. This thermal event was observed in the temperature range of 215–221 K. In the literature, there are several works reporting the T_g_ of GL in a temperature range similar to that reported herein [26,27,29,31,32,40]. However, the complex thermal transition (T_g_, and T_dv_) observed in GL has not been reported so far, mainly because it is difficult to differentiate two or more events by conventional DSC techniques. The vitrification process is the phenomenon occurring during the cooling of a liquid below its T_g_, where the disordered structure of the liquid remains in the solidified glass [41]. The purpose of vitrification of liquids is avoiding the formation of ice crystals during cooling. By contrast, devitrification (T_dv_) is the process occurring during the heating of a glass above its T_g_, and the subsequent formation of crystals. Boyer, Heeschen and Gilham [42] reported the supercooling of GL determined by nuclear magnetic resonance (NMR) in the temperature range of 213.15–223.15 K. They related this thermal event to the molecular dynamic changes of the system as a function of the orientation, and the dipole interactions among the neighbor molecules. Sou et al. (2011), employed ultrasensitive calorimetry, and observed an exothermic peak in the range of 210.15–250.15 K; this event was associated with the crystallization of GL. The ultrasensitive calorimetry detects heat flow signals in the order of the 10^−9^ watts, and employs very low heating rates of 0.005–4 mK/s. At these heating conditions, it is likely that the system may have enough time to pass from a metastable state such as the amorphous state, into a state of greater energy such as the crystalline state. In addition to the instrumental conditions exerted, the purity of the GL may influence the results of the several thermal events.

**Figure 4:**
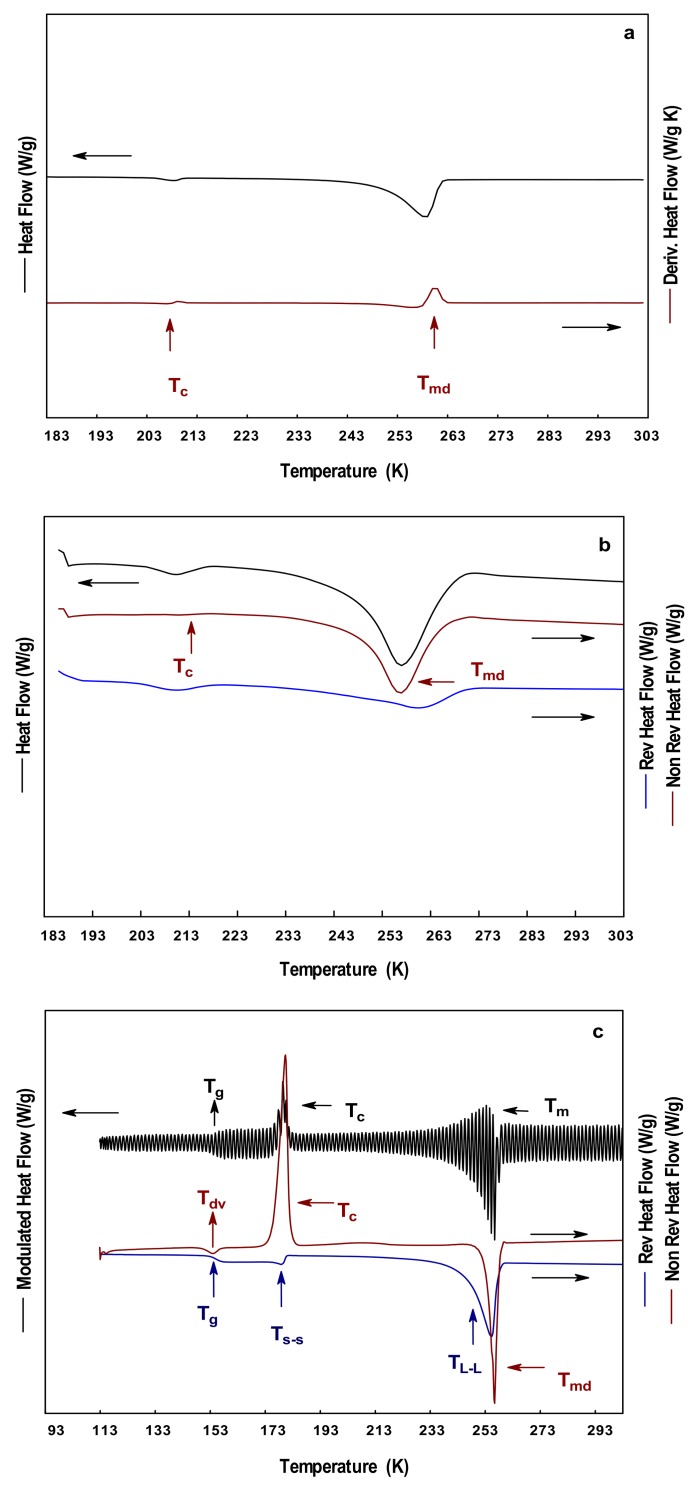
Thermograms of EG at 278 K/min: (**a**) DSC; (**b**) MDSC; and (**c**) SMDSC.

Figure 4 shows the results of the thermal analysis carried on the EG at a heating rate of 278 K/min. The total heat curve from both DSC and MDSC (Figure 4a,b, respectively) showed two endothermic events at about 203–215 K and 234–268 K. However, other thermal events were observed in the MDSC curves. The separation of the signals from the MDSC allowed the identification of these thermal events. The non-reversible heat flow curve showed only an endothermic peak corresponding to the melting of EG in a temperature of 255 K. The reversible heat flow curve showed two events associated with a solid–solid transition (T_S-S_) at 203–215 K, and a liquid–liquid transition (T_L-L_) about 261–286 K. When the temperature was decreased to 113 K in the S-MDSC, more thermal events were observed (Figure 4c). The modulated heat flow curve showed three events identified as the glass transition temperature, crystallization temperature, and melting temperature. However, the decomposition of the signal evidenced that these events were complex, since they did not appear alone. The non-reversible heat flow curve showed an endothermic peak at 154 K identified as the devitrification of EG, an exothermic peak at 175 K associated to the crystallization, and an endothermic peak at 255 K related to the melting of EG. The reversible heat flow curve showed the stepped change in the slope about 154 K corresponding to the T_g_, a solid–solid transition occurred at 178 K, and an endothermic peak at 255 K identified as a liquid–liquid transition. The T_m_ reported in this work was in the order of those values reported for mixtures of EG and water [43,44], while other thermal events agreed with those determined with other characterization techniques [43,45,46].

**Figure 5:**
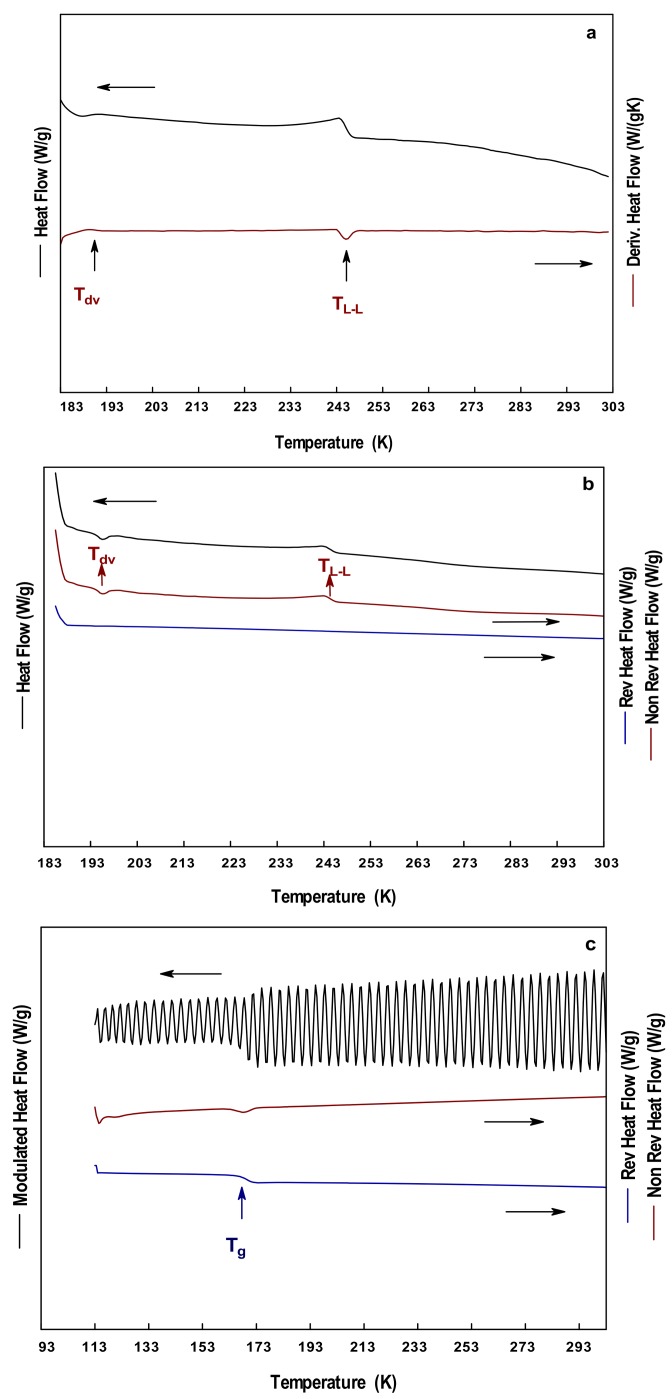
Thermograms of PG at 278 K/min: (**a**) DSC; (**b**) MDSC; and (**c**) SMDSC.

Figure 5 shows the thermograms of the PG obtained at a heating rate of 278 K/min. The total heat flow curve showed in both curves, DSC and MDSC (Figure 5a,b, respectively), the presence of two thermal events at 193–245 K. The separation of the signal in the MDSC showed that these signals were non-reversible, thus were dependent on time and temperature. The signals were identified as the devitrification at 193 K, and as a liquid–liquid transition at 245 K. The devitrification of PG has been reported in the range of 193–203 K [47,48]. The further decrease in the temperature by S-MDSC showed a complex thermal event in the range of 163–171 K (Figure 5c). The event was identified as the glass transition temperature in the reversible heat flow curve at 167 K, and as a devitrification event in the non-reversible heat flow curve. Other thermal events such as crystallization and melting were not observed during the experiments. This suggested that either the cooling temperature (113 K) was not sufficient to induce the formation of crystals or the PG remained as an amorphous solid (i.e., uncrystallized) at that temperature. Fahy [49] indicated the importance of knowing the T_g_ of PG during the cooling process, and described the vitrification process as the increment in the viscosity of the liquid. During the vitrification, the liquid transforms into a glass, where the molecular movements are significantly restricted. Other works have employed mixtures of PG, and soy protein to determine the T_g_ of PG [50].

Table 2 summarizes the different thermal events observed during the study of the polyols by DSC, MDSC, and S-MDSC.

In pharmaceutical formulations, the use of polyols may depress the glass transition temperature of the system in the range of 213–213 K for non-aqueous solvents, and below 348 K for aqueous systems [51,52]. This is important in the field of drug release where the releasing temperature may be modified by the addition of a plasticizer [53]. Polyols have also been employed as an aid in the differentiation of complex thermal events in water [54]. Water present a liquid–liquid transition together with the formation of ice at 235 K. Thus, to separate these events, several mixtures of water–glycerol, water–1,2,4-butanetriol, and water–AlCl_3_ have been tested. Polyols have been extensively employed in the cryopreservation of human sperm [55]. During the cooling, crystallization of water is unwanted since the sharp morphology of water crystals may damage the cell wall of the sperm. Then, it is desirable to reach the vitreous state through the vitrification process.

## 4. Conclusions

This study contributes to the characterization of the thermal properties of polyols. The thermal events of three polyols were determined by direct characterization techniques such as simultaneous thermogravimetric analysis and differential thermal analysis (TGA-DTA), differential scanning calorimetry (DSC), modulated DSC (MDSC), and super cooling MDSC (S-MDSC). TGA-DTA allowed to properly distinguishing the boiling temperature range of the polyols. Complex thermal events were identified with the aid of modulated DSC techniques, observing that the glass transition temperature (T_g_) was accompanied by the devitrification (T_dv_) of the glassy liquid. The T_g_ value of the polyols was determined as 191, 154, and 167 K for glycerol, ethylene glycol, and propylene glycol, respectively. Additionally, other thermal events such as liquid–liquid (T_L-L_) and solid–solid (T_S-S_) transitions were observed as slight changes in the slope of the non-reversible heat flow curve. Evidently, the use of the MDSC and SMDCS allowed separating the contributions dependent on time and temperature (non-reversible heat flow) from those dependent on the heat capacity (reversible heat flow). Although glycerol is the polyol most often employed as plasticizer, the characterization of the thermal properties of other polyols such as ethylene glycol and propylene glycol enlarge the window for new potential applications as plasticizers in the food industry.

## Figures and Tables

**Table 1:** Boiling temperature range of the polyols, determined by simultaneous TGA-DTA at different heating rates.

		Heating Rate (K/min)					
		TGA			DTA		
		275	278	283	275	278	283
**GLY**	Mass loss (%)	373 ± 1.4	373 ± 7.9	373 ± 2.9	-	-	-
	Initial mass loss temperature (K)	313 ± 2.8	398 ± 5.6	407 ± 3.1	374 ± 0.6	393 ± 2.1	412 ± 2.7
	Maximum mass loss temperature (K)	483 ± 1.6	502 ± 4.3	522 ± 2.4	488 ±1.2	506 ± 3.8	529 ± 5.6
	Final mass loss temperature (K)	495 ± 3.5	511 ± 5.1	538 ± 5.1	493 ± 3.4	531 ± 2.4	552 ± 6.1
**EG**	Mass loss (%)	373 ± 7.1	373 ± 2.5	373 ± 4.3	-	-	-
	Initial mass loss temperature (K)	331 ± 4.7	338 ± 3.7	345 ± 4.8	332 ± 2.0	338 ± 0.8	345 ± 2.1
	Maximum mass loss temperature (K)	410 ± 6.3	427 ± 1.4	442 ± 3.8	413 ± 2.8	433 ± 0.4	457 ± 1.8
	Final mass loss temperature (K)	417 ± 2.1	442 ± 3.1	457 ± 3.3	425 ± 1.4	459 ± 1.7	496 ± 1.5
**PG**	Mass loss (%)	373 ± 4.6	373 ± 3.9	373 ± 4.5	-	-	-
	Initial mass loss temperature (K)	323 ± 2.3	333 ± 2.7	334 ± 7.7	325 ± 3.4	333 ± 2.8	403 ± 6.1
	Maximum mass loss temperature (K)	401 ± 2.8	418 ± 1.0	431 ± 0.6	404 ± 4.0	422 ± 4.8	436 ± 5.0
	Final mass loss temperature (K)	410 ± 0.9	429 ± 2.1	444 ± 0.4	416 ± 2.9	448 ± 3.5	480 ± 1.0

**Table 2:** Summary of the identification of the thermal events of polyols, and the temperature at which they occurred.

Polyol	Thermal Event (K)						
	T_g_	T_dv_	T_c_	T_s-s_	T_L-L_	T_m_	T_B_
GL	191–199	197	-	-	215–221	-	513.15
EG	154	154	175	178	255	255	437.15
PG	167	193	-	-	245	-	431.15
